# Kinetic modelling of ultrasound-triggered chemotherapeutic drug release from the surface of gold nanoparticles

**DOI:** 10.1038/s41598-023-48082-9

**Published:** 2023-12-02

**Authors:** Tyler K. Hornsby, Farshad Moradi Kashkooli, Anshuman Jakhmola, Michael C. Kolios, Jahangir (Jahan) Tavakkoli

**Affiliations:** 1 Department of Physics, Toronto Metropolitan University, Toronto, Canada; 2 Institute for Biomedical Engineering, Science and Technology (iBEST), Li Ka Shing Knowledge Institute, St. Michael’s Hospital, Toronto, Canada

**Keywords:** Drug delivery, Chemotherapy, Targeted therapies

## Abstract

Therapeutic ultrasound can be used to trigger the on-demand release of chemotherapeutic drugs from gold nanoparticles (GNPs). In the previous work, our group achieved doxorubicin (DOX) release from the surface of GNPS under low-intensity pulsed ultrasound (LIPUS) exposure. However, the specific release kinetics of ultrasound-triggered DOX release from GNPs is not known. Here, we present a release kinetics study of DOX from GNPs under ultrasound exposure for the first time. A novel dialysis membrane setup was designed to quantify DOX release from LIPUS-activated GNPs at 37.0 °C and 43.4 °C (hyperthermia temperature range). Contributions of thermal and non-thermal mechanisms of LIPUS-triggered DOX release were also quantified. Non-thermal mechanisms accounted for 40 ± 7% and 34 ± 5% of DOX release for 37.0 °C and 43.4 °C trials, respectively. DOX release under LIPUS exposure was found to follow Korsmeyer–Peppas (K–P) kinetics, suggesting a shift from a Fickian (static) to a non-Fickian (dynamic) release profile with the addition of non-thermal interactions. DOX release was attributed to an anomalous diffusion release mechanism from the GNP surface. A finite element model was also developed to quantify the acoustic radiation force, believed to be the driving force of non-thermal DOX release inside the dialysis bag.

## Introduction

In conventional chemotherapy, the non-specific nature of chemotherapeutic drugs can lead to side effects impairing quality of life and, in some cases, even lasting years after treatment^1^. To overcome the toxicity issues present with conventional chemotherapy, chemotherapeutic drugs can be coupled with nanoparticle drug carriers to improve trial efficacy and safety, as nanoparticle drug carriers allow for protection from premature drug activation and accumulation at the tumour site via the enhanced permeability and retention effect^2,3^. Furthermore, external stimuli, such as ultrasound waves, can trigger chemotherapeutic drug release from nanoparticle drug carriers at the tumour site while sparing surrounding healthy tissue and organs at risk^3–6^.

Many nanoparticle drug carriers are available for chemotherapeutic drugs; however, gold nanoparticle (GNP) drug carriers, in particular, have been proven advantageous due to their customizable size and shape, inertness, and low toxicity^7^. GNPs can be easily synthesized in the < 10 nm range and functionalized with various anticancer drugs^8,9^. As a result, GNPs already have FDA approval as anticancer drug carriers in clinical trials^10^. GNPs have also shown stand-alone anticancer properties by enhancing apoptosis in leukemia cells and inhibiting proliferation in various cancer cell lines^11,12^. GNP cytotoxicity appears highly dependent on particle size^13^; however, GNPs could provide a synergistic anticancer effect when combined with chemotherapeutic drugs as drug delivery vehicles. GNPs have also shown potential as sonosensitization agents under therapeutic ultrasound exposure. In a study by Beik et al.^14^, the sonosensitizer properties of GNPs were demonstrated in an in vivo animal study of colorectal tumour-beating mice. Here, the mice were intraperitoneally injected with GNPs before being subjected to therapeutic ultrasound exposure, which saw a significant reduction in tumour volume^14^. Further support for GNPs as sonosensitizers has also been presented by Shanei et al.^15^ in an in vitro model of HeLa cervical cancer cells, where therapeutic ultrasound and radiation therapy were used in combination with GNPs to observe a synergistic anticancer effect. Overall, GNPs are an attractive option for ultrasound-triggered nanoparticle chemotherapeutic drug delivery.

Previous studies have reported that when delivering drug-loaded GNPs intravenously, ≥ 90% of GNPs may remain in the blood circulation for 7 days or more, before accumulating primarily in the liver, kidney and spleen^16,17^. Small GNPs (< 5–8 nm in diameter) are believed to undergo rapid renal clearance, while excretion of larger GNPs is primarily done by a combination of complex processes in the liver, thus avoiding long-term GNP accumulation-associated toxicity^18–20^. GNPs in the size range of 10–250 nm can be cleared from the body, preventing accumulation-associated toxicity^20,21^. This is believed to be achieved through the hepatobiliary system^22^ by a complex combination of processes, which include the cellular exocytosis of the nanoparticles internalized in healthy tissue cells and the mononuclear phagocytic system of the liver^23^. In in vivo GNP toxicity studies, GNPs were found to accumulate in tissue proportionally with dose, and even after repeated administration, did not produce any mortality or any indication of toxicity^17^.

When designing nanoparticle drug delivery systems, the general workflow is as follows: (1) formulation of nanoparticle drug carrier and chemotherapeutic drug design, (2) conducting drug release trials, (3) mathematical analysis of drug release measurements, and (4) formulation of specific release kinetics^24^. The mathematical analysis of drug release and specific release kinetics is important in this process as it provides insight into the complex dynamics of drug release profiles and mechanisms of release^24–26^ and allows for optimization of the drug delivery system before being utilized in an in vivo model^27^. Several mathematical models are available to depict drug release kinetics; the zero-order, first-order, Higuchi, and Korsmeyer–Peppas (K–P) are the most common^25,26,28,29^. Additionally, kinetics models can be combined and modified for more complicated drug release profiles, as seen with biodegradable nanoparticle drug carriers^30^. These models can be fitted to drug release data, and the degree of agreement of the fit with each model can be used to make conclusions on drug release mechanisms and quantify release constants^26^.

England et al.^31^ found that cisplatin and paclitaxel release from layered GNPs in a dialysis membrane experiment best agreed with the Higuchi and K–P kinetics models. Since both models describe drug release from degrading matrix and polymer systems, they concluded that the multi-layered GNPs could be modeled similarly to a system which undergoes degradation^31^. In work by Thambiraj et al.^32^, docetaxel release from citrate-capped GNPs inside a dialysis membrane was also studied and found to show good agreement with Higuchi kinetics. Here, docetaxel release from GNPs was observed following an initial burst release phase, a second sustained release phase due to Fickian diffusion across the dialysis membrane, and a third slow constant release phase due to continued release from the GNPs^32^. Based on the agreement with Higuchi kinetics, it was concluded that the docetaxel release was attributed to diffusion and erosion mechanisms^32^. In ultrasound-triggered nanoparticle drug delivery systems, kinetic modelling has also been explored for liposome drug carriers^33,34^. Typically, these studies have found some degree of success with fitting the K–P kinetics model; however, to the best of our knowledge, there has been no proposed kinetic modelling of ultrasound-triggered drug release from GNP drug carriers.

In the previous works by our group^35,36^, we have developed and tested an ultrasound mediated GNP drug delivery system, with a patented low-intensity pulsed ultrasound (LIPUS) prototype device^37^, for use with a conventional chemotherapeutic drug, doxorubicin (DOX). This drug delivery system achieved LIPUS-induced DOX release in an *ex vivo* tissue model for a fixed 5-minute LIPUS trial. Here LIPUS exposure was sufficient to heat the region of interest to the therapeutic hyperthermia temperature regime (temperature range of 41–45 °C), which has been shown to improve drug uptake into solid tumours by increasing blood flow, perfusion rate, and spacing in endothelial junctions^38^. The LIPUS-induced thermal and non-thermal (mechanical) contributions to DOX release were also isolated and quantified in the ex vivo model^8,39^. It was found that non-thermal LIPUS contributions to DOX release were significant and driven by the acoustic radiation force (ARF) of the LIPUS field^8^. However, DOX release kinetics from GNPs under LIPUS exposure are still not well understood.

In the current work, DOX release kinetics from GNP drug carriers were evaluated using a dialysis membrane method commonly used for determining drug release kinetics from nanocarrier systems. This work marks the first drug release kinetics study for GNPs under ultrasound exposure. Released DOX was collected from the sink receiver compartment for two different trials, including (1) water bath heating trials, where DOX release is attributed to thermal interactions only, and (2) LIPUS trials, where DOX release is attributed to a combination of both thermal and non-thermal interactions. The two LIPUS trials were designed to mimic the fixed 5-min exposure available with our prototype LIPUS device. DOX release for both trials was compared to quantify the contributions of LIPUS thermal and non-thermal drug release mechanisms using weighting factor calculations established in our previous ex vivo model^8^. DOX release was measured as a function of time for all trials, and the data was fitted with zero-order, first-order, Higuchi and K–P common kinetic models. This study explored the mechanisms responsible for DOX release from the GNP surface under LIPUS exposure. Lastly, a finite element model was developed to quantify LIPUS ARF inside the dialysis bag, as ARF is believed to be a driving force in LIPUS-induced drug release from GNPs^8^.

## Methods

### Synthesis of DOX-loaded GNPs

All DOX-loaded GNPs were synthesized using a modified green synthesis method based on Jakhmola et al.^35^. Briefly, 0.5 mL of trisodium citrate solution (38.8 mM) and 20 μL of aqueous 10mM DOX were mixed in a 1.5 mL centrifuge tube and sonicated (1510 Ultrasonic Cleaner, Branson Ultrasonics, Brookfield, CT) to yield a homogenous, orange-colored solution. This was followed by the addition of a 0.5 mL solution of chloroauric acid (4 mM). The final concentrations of reactants in the reaction mixture were chloroauric acid (2.0 mM), trisodium citrate (19.4 mM) and DOX (0.2 mM). Gold ions were then reduced slowly by citrate at room temperature until a red colloidal sol of spherical GNPs formed in under an hour. This yields 7.4 ± 0.5 nm diameter spherical GNPS with trisodium citrate and DOX bound noncovalently to the surface^27^. Lastly, the solution was centrifuged and resuspended in MilliQ® water to remove any unreacted reactants. The amount of DOX-loaded onto the GNPs was determined indirectly by measuring un-bound DOX fluorescence in the supernatant. It was observed that the supernatant was colorless and displayed negligible fluorescence compared to the fluorescence of the initial DOX concentration of 0.2 mM. Therefore, we concluded that the concentration of the loaded DOX was 0.2 mM. More details of the synthesis method, mechanism and GNP characterization are provided in^8,9,27^. Gold(III) chloride trihydrate (99.9%) and trisodium citrate dihydrate were purchased from Sigma Aldrich (St. Louis, MO, USA). DOX hydrochloride salt (> 99%) was purchased from LC Laboratories (Woburn, MA, USA). MilliQ® water was used in all the synthesis and dialysis experiments (Milli-Q® Integral water purification system, Sigma Aldrich). Structural characterization of the DOX-loaded GNPs was performed with a transmission electron microscope (Hitachi HT7800) operating at 300 kV. Additional characterization of the DOX-loaded GNPs before and after LIPUS exposure is available in our previous works^8,27^.

### DOX release trials

**Figure 1:**
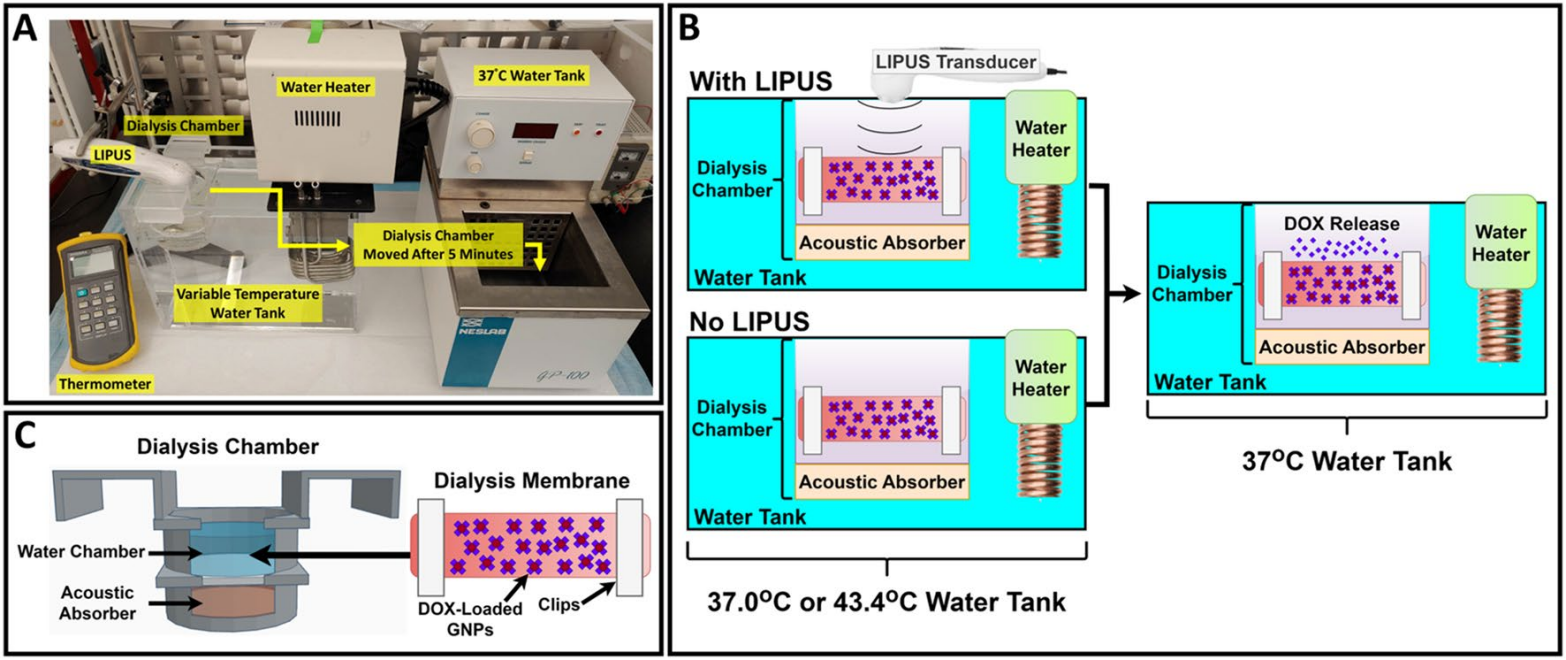
(**A**) A picture of the dialysis membrane DOX release setup. (**B**) A schematic of the with LIPUS and without LIPUS DOX release procedures. Here, the dialysis chamber is submerged in a 37.0 °C or 43.4 °C water bath with or without LIPUS for five minutes, followed by 55 min in a fixed 37 °C water bath. (**C**) A schematic of the dialysis chamber and the dialysis membrane containing DOX-loaded GNPs. The dialysis membrane is submerged in the isolated water chamber volume for all release trials.

The dialysis membrane setup is shown in Fig. 1A. For all DOX release experiments, a 1.7 mL solution of DOX-loaded GNPs was pipetted into a 5.4 cm length and 6.4 mm diameter 12–14 kD molecular weight cutoff dialysis bag (Spectra/Por 2 Dialysis Trial Kit, Spectrum Laboratories, Rancho Dominguez, CA). This dialysis bag was selected because it is permeable to DOX and not GNPs^31,32^. A custom-made cylindrical 7.4 cm diameter acrylic dialysis chamber was constructed to suspend the dialysis bag in an enclosed 150 mL of purified water. An acoustic absorber was positioned at the bottom of the dialysis chamber and attached via a 0.1 mm thick ultrasound transparent film. The dialysis chamber was then fixed to a variable temperature water tank, with the dialysis chamber volume kept isolated from the outside tank volume. The dialysis chamber was designed to be removed from the variable water tank, while keeping the 150 mL water volume and dialysis membrane isolated and contained. A prototype handheld LIPUS transducer with 8.4 W power, 50% duty cycle, 1 kHz pulse repetition frequency and a fixed insonation time of 5 min was then attached to the top of the dialysis chamber. A Haake™ water heater (Haake DC 10 Thermo Controller 003-2859, ThermoFisher Scientific, Waltham, MA, USA) was used to control the water tank’s temperature with a built-in temperature controller. The temperature inside the dialysis chamber was monitored throughout all experiments using an Omega™ thermometer (HH309A Four-Channel Data Logger, Omega Engineering, Norwalk, CT).

The dialysis bag containing the DOX-loaded GNP sample was suspended in the dialysis chamber. Five-minute DOX release trials were performed as follows: (1) the dialysis chamber was placed in the variable temperature water tank without LIPUS exposure (thermal release), and (2) the dialysis chamber was placed in the variable temperature water tank with LIPUS exposure at 8.4 W and 50% duty cycle (thermal and non-thermal release). The LIPUS settings used to trigger DOX release from the surface of GNPs were determined in previous parametric studies performed by our group in^8,27^. Both release trials were performed for variable water bath temperatures of 37.0 °C and 43.4 °C, yielding four trials in total. A diagram of the DOX release procedure is provided in Fig. 1B, and a schematic of the dialysis chamber is provided in Fig. 1C. A trial temperature of 37.0 °C was selected as a clinically relevant temperature, and 43.4 °C was selected as a typical hyperthermia temperature that the LIPUS transducer can heat ex vivo tissue. After each 5-min release trial, the entire dialysis chamber (Fig. 1C) was removed from the water bath and placed in a second fixed 37.0 °C water bath (Neslab GP-100, Neslab Instruments, Newington, NH) for 55 min to allow transport of DOX across the dialysis membrane. A 1.5 mL sample was extracted from the dialysis chamber volume immediately after the 5-min trial, and 1.5 mL of purified water was added to keep the volume constant. This was repeated in 10-min intervals for the remainder of each trial in the secondary water bath. To monitor the temperature profile inside the dialysis membrane during DOX release, experiments were also performed for thermal release (no LIPUS) at 37.0 °C and 43.4 °C with a thermocouple secured to the surface of the dialysis bag. All DOX release trials were repeated six times (N = 6).

To quantify DOX release, the fluorescence of the extracted samples was measured using a spectrofluorophotometer (RF-5301 spectrofluorophotometer, Shimadzu, Kyoto, Kyoto, Japan). Cumulative DOX release (${Q}_{t}$) was then calculated as a percent value as follows^40^:

1 $${Q}_{t} \left(\%\right)=\frac{{{I}_{t}-I}_{0}}{{I}_{max}-{I}_{0}}\times 100\%$$

Here ${I}_{t}$ is DOX fluorescence intensity at time $t$, ${I}_{0}$ is the baseline fluorescence intensity, and ${I}_{max}$ is the maximum fluorescence intensity. In this work, the baseline intensity was set to the first measurement in the no LIPUS at 37.0 °C trial (the lowest measured DOX release value), and ${I}_{max}$ was set to the final measurement with LIPUS at 43.4 °C trial (the highest measured DOX release value). The calculated ${Q}_{t}$ value was used to represent cumulative percent DOX release when fitting zero-order, Higuchi, and K–P kinetics models. When using the first-order kinetics model, the percent remaining DOX (${Q}_{R}$) was calculated as follows:

2 $${Q}_{R} \left(\%\right)=100-{Q}_{t}$$

### Kinetic modelling of DOX release

To study release kinetics, DOX release for all four trials was evaluated with zero-order (Eq. 3), first-order (Eq. 4), Higuchi (Eq. 5), and K–P (Eq. 6) kinetics models as follows^25,26^:

3 $${Q}_{t}={K}_{0}t$$

4 $$\mathrm{log}\left({Q}_{R}\right)=\mathit{log}\left({Q}_{0}\right)-\frac{{K}_{1}t}{2.303}$$

5 $${Q}_{t}={K}_{H}\sqrt{t}$$

6 $${Q}_{t}={K}_{KP}{t}^{n}$$

where ${Q}_{t}$ is the amount of DOX released at time $t$, ${Q}_{0}$ is the amount of initial DOX in the dialysis chamber, $n$ is the release exponent, and ${K}_{0}$, ${K}_{1}$, ${K}_{H}$ and ${K}_{KP}$ are the zero-order, first-order, Higuchi, and K–P release constants, respectively.

To evaluate the zero-order kinetics, cumulative DOX release *vs*. time was plotted, and a linear fit was applied with ${K}_{0}$ as the slope. First-order kinetics were evaluated by plotting the log of remaining DOX *vs.* time and applying a linear fit with ${- \, K}_{1}/2.303$ as the slope. Higuchi kinetics was evaluated by plotting cumulative DOX release *vs.* the square root of time and applying a linear fit with ${K}_{H}$ as the slope. Lastly, K–P kinetics were evaluated by plotting the log of cumulative DOX release versus the log of time and applying a linear fit with $n$ as the slope and $log({K}_{KP})$ as the y-intercept^25^. All linear fitting was performed using the MATLAB R2023a (MathWorks, Natick, MA, USA) curve fitting tool with a 1st order polynomial least absolute residuals robust fit. Common metrics for agreement of fit with each kinetics model were then calculated, including the adjusted coefficient of determination (${R}_{a}^{2}$), root mean square error (RMSE) and sum of square errors (SSE)^41,42^.

### Quantifying thermal and non-thermal release contributions

To quantify the contribution of thermal and non-thermal mechanisms of LIPUS-induced DOX release in this study, thermal (${\omega }_{T}$) and non-thermal (${\omega }_{NT}$) weighting factors were calculated using the equations presented in^8^ for all 37.0 °C and 43.4 °C trials as follows:

7 $${\omega }_{T}\left(\%\right)=\frac{{I}_{max}\left(No\,LIPUS\right)}{{I}_{max}\left(LIPUS\right)}\times 100$$

8 $${\omega }_{NT}\left(\mathrm{\%}\right)=\frac{{I}_{max}\left(LIPUS\right)-{I}_{max}\left(No\,LIPUS\right)}{{I}_{max}\left(LIPUS\right)}\times 100$$

where ${I}_{max}\left(No\,LIPUS\right)$ and ${I}_{max}\left(LIPUS\right)$ are the maximum fluorescence intensity of released DOX for trials without and with LIPUS, respectively.

### Development of an acoustics simulation model

**Figure 2:**
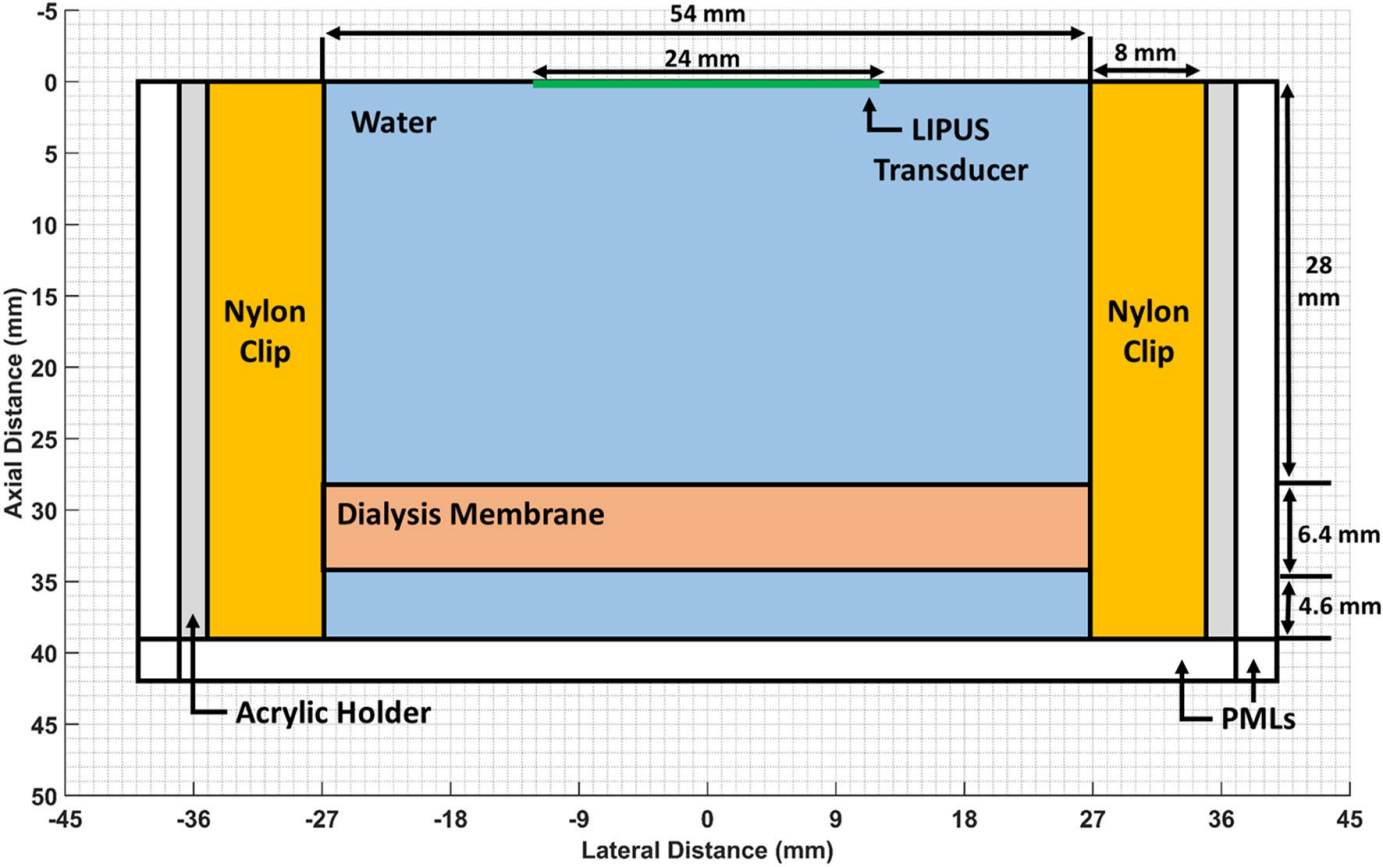
The 2D axisymmetric geometry used for COMSOL simulation in this study. The LIPUS piston transducer is defined as a flat line, while the water, dialysis membrane, nylon clip, and acrylic holder domains are labelled for reference. The bottom of the dialysis chamber setup is an acoustic absorber and defined as a PML. PMLs are also set on the outside of the dialysis chamber walls, as no significant reflection from outside the chamber is considered.

To visualize the LIPUS acoustic field inside the dialysis membrane and quantify the ARF of the ultrasound field, the dialysis membrane setup was simulated using a 2D axisymmetric geometry on COMSOL 6.0 (COMSOL Multiphysics Modeling Software, Stockholm, Sweden). A 3 mm wide perfectly matched layer (PML) was applied to the boundaries of the computational domain to prevent LIPUS beam reflection. A free triangular mesh was generated throughout the geometry with a maximum mesh size of 1/60th the LIPUS wavelength. Note that a fine mesh size was selected as computing time was not a limitation in this study. A diagram of the COMSOL geometry is provided in Fig. 2, and the main COMSOL parameters are presented in Table 1.

**Table 1:** Main material and LIPUS parameters used in the COMSOL simulation.

Domain	Parameter	Value	Unit
LIPUS Transducer	Frequency	1.0	MHz
	Acoustic Power	8.4	W
	Duty Cycle	50%	–
	Transducer Diameter	24	mm
Water	Attenuation Coefficient	0.0022	dB/(cm MHz^2^)
	Density	994.23	kg/m^3^
	Speed of Sound	1520.6	m/s
	Absorption Coefficient	0.0253	Np/m
Acrylic Holder	Density	1190	kg/m^3^
	Young's Modulus	3.2	GPa
	Poisson's Ratio	0.35	–
Nylon Clips	Density	1150	kg/m^3^
	Young's Modulus	2.0	GPa
	Poisson's Ratio	0.4	–

Material parameters were taken from^57,58^.

To generate the LIPUS pressure and intensity fields, the axisymmetric Helmholtz equation was solved using COMSOLs pressure acoustics module in the frequency domain, with an inward displacement applied to the LIPUS transducer face^27^. The acrylic holder and nylon clip domains were simulated using COMSOLs solid mechanics module, with the water domain boundary defined using acoustic-structure interaction multiphysics. Both the ARF and mechanical index (MI) were then calculated inside the dialysis membrane as follows^43,44^:

9 $$\mathrm{ARF}=\frac{2\alpha I}{c}$$

10 $$\mathrm{MI}=\frac{{P}_{-}}{\sqrt{f}}$$

where $\alpha$ is the absorption coefficient of water (Np/m), $I$ is time-averaged intensity in the dialysis bag (W/cm^2^), $c$ is the speed of sound in water (m/s), ${P}_{-}$ is the negative peak pressure in the dialysis bag (MPa), and $f$ is the center frequency of the LIPUS device (MHz).

## Results

### Synthesis of DOX-loaded GNPs

**Figure 3:**
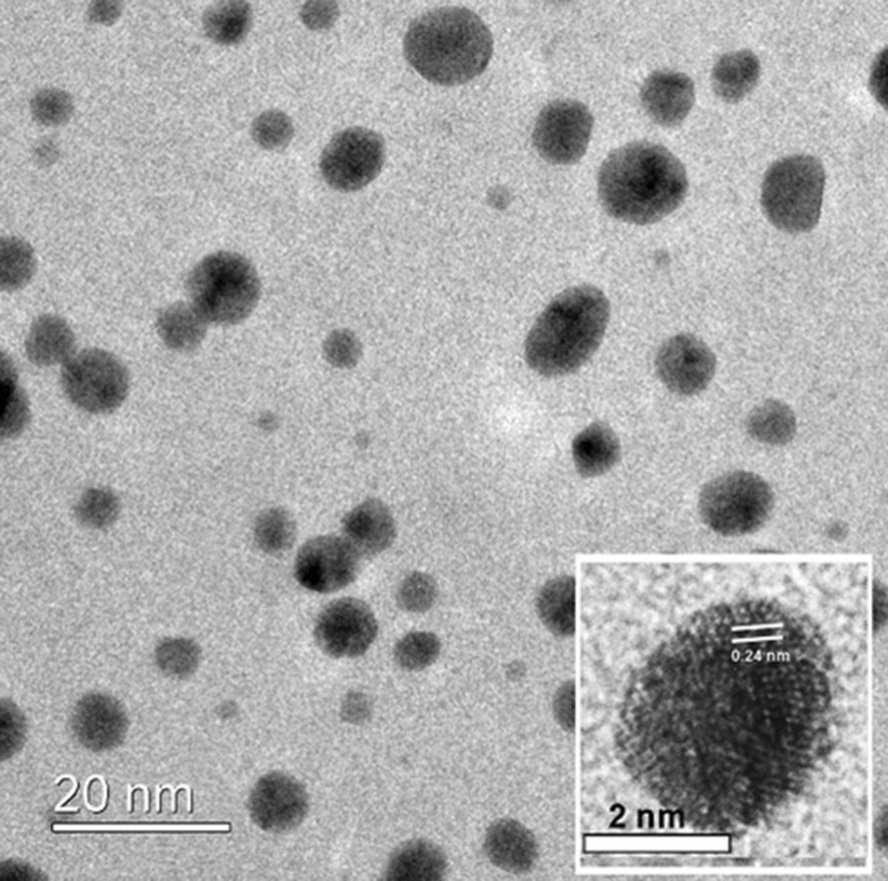
High-resolution transmission electron microscopy micrographs of the DOX-loaded GNPs. GNPs in the size range of 7–9 nm are visible, with the 2. 4 Å lattice fringe spacing marked in the inlet for reference.

High-resolution transmission electron microscopy micrographs of the DOX-loaded GNPs recorded spherical particles with a size range of 7–9 nm (Fig. 3). The images displayed multiple crystalline domains in the GNPs with a lattice fringe spacing of about 2.4 Å. The GNPs were polycrystalline, with a high concentration of defects, dislocations and stacking faults, and contained many ultrafine crystalline Au grains with disordered areas surrounding them^45^. Here, DOX acts as the shape-directing molecule, as in the absence of DOX, a black-colored colloidal solution is formed with a nanowire network morphology^46^.

### DOX release studies

**Figure 4:**
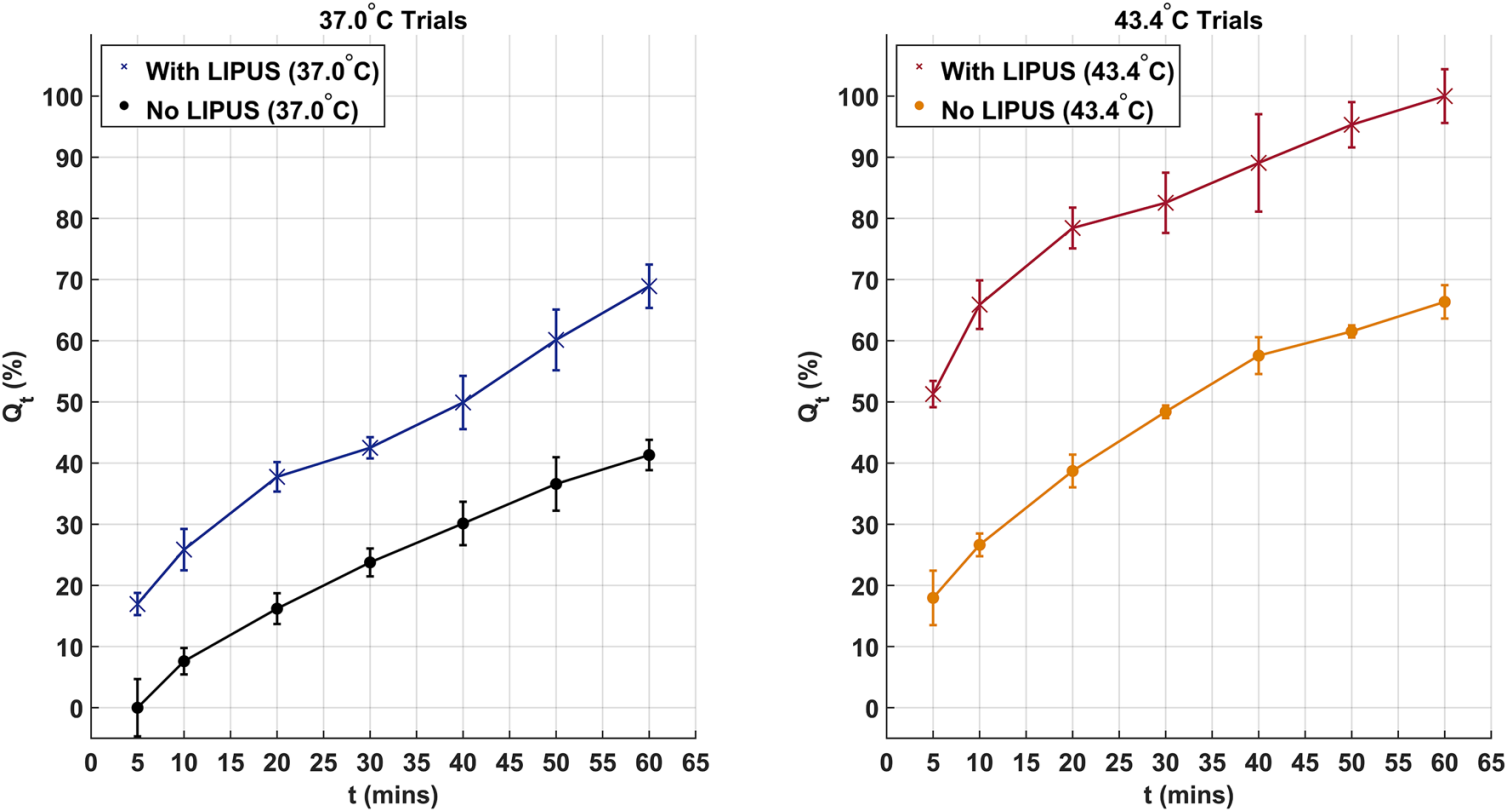
(**A**) Measured DOX release for trials with and without LIPUS at 37.0 °C. (**B**) Measured DOX release for trials with and without LIPUS at 43.4 °C. All DOX release values are averaged over six repeated experiments (N = 6), with uncertainty as the standard error.

**Figure 5:**
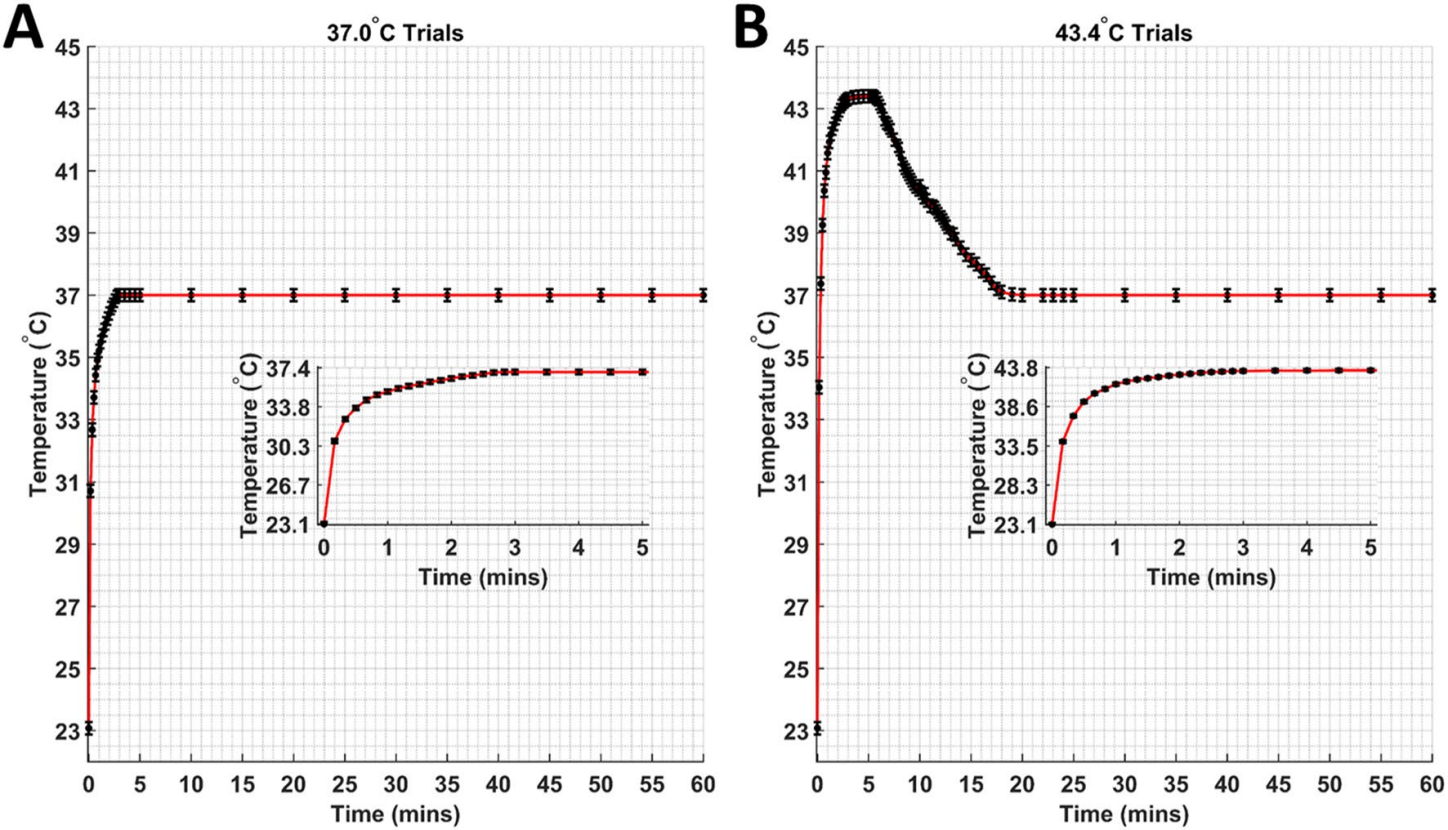
Dialysis membrane surface temperature for (**A**) trials without LIPUS at 37.0 °C, and (**B**) trials without LIPUS at 43.4 °C. All temperature values are averaged over six repeated experiments (N = 6), with uncertainty taken as the average standard error. For both temperatures, an inset depicting the temperature profile for the first 5-min is provided.

Measured DOX release for both 37.0 °C trials is provided in Fig. 4A, while DOX release for both 43.4 °C trials is provided in Fig. 4B. Measured temperature profiles at the surface of the dialysis membrane are provided in Fig. 5. The DOX-loaded GNP insertion into the dialysis membrane was performed at room temperature (23.1 °C) for all DOX release experiments (37.0 °C and 43.4 °C with and without LIPUS). The assumption was that the average temperature inside the dialysis membrane was approximately equal to the surface temperature. Additionally, no change in the temperature profile due to LIPUS absorption was considered due to the low attenuation coefficient of water and low time-averaged intensity of the LIPUS beam^47^. In the 37.0 °C trials (Fig. 5A), the dialysis bag temperature reached 37.0 °C 2.83 min after the start of the trial and remained constant for the duration of the trial. In the 43.4 °C trials (Fig. 5B), the dialysis bag temperature reached 43.4 °C 3.5 min after the start of trial, then cooled to 37.0 °C 13 min post-exposure after being placed in the secondary 37 °C water bath. The dialysis membrane temperature profile seen in the 43.4 °C trial is comparable to that planned for in an in vivo application. The 5-min LIPUS exposure would first heat the GNPs to the hyperthermia temperature regime, then the LIPUS transducer would be turned off, and the GNPs would be allowed to cool to the background 37 °C temperature.

### Kinetic modelling of DOX release

Zero-order, first-order, Higuchi, and K–P kinetics models were fitted to the measured DOX release data presented in Fig. 4. The Higuchi, K–P, and First-order kinetic models agreed well with the thermal release trials (no LIPUS). However, the Higuchi kinetics model showed the best agreement with thermal release overall. Both the K–P and First-order kinetic models showed high agreement with LIPUS release trials. However, the K–P kinetic model showed the best agreement. Calculated ${R}_{a}^{2}$, RMSE, SSE and release coefficients for all kinetic fits are provided in Table 2. Fit agreement for trials with and without LIPUS was quantified by averaging ${R}_{a}^{2}$ over both temperatures tested and not considering fits with high RMSE or SSE.

**Table 2:** Calculated release constants, RMSE, SSE and average adjusted R-squared values for each kinetic model fitted to the DOX release data.

Trial	Zero-order	First-order	Higuchi	K–P
No LIPUS (37.0 °C )	${K}_{0}=0.725$ $RMSE=2.342$ $SSE=27.41$	${K}_{1}=0.021$ $RMSE=0.016$ $SSE=0.001$	${K}_{H}=7.498$ $RMSE=0.536$ $SSE=1.436$	${K}_{KP}=1.13$ $RMSE=0.074$ $SSE=0.022$
No LIPUS (43.4 °C )	${K}_{0}=0.872$ $RMSE=4.154$ $SSE=86.27$	${K}_{1}=0.037$ $RMSE=0.037$ $SSE=0.007$	${K}_{H}=8.927$ $RMSE=1.432$ $SSE=10.25$	${K}_{KP}=7.69$ $RMSE=0.031$ $SSE=0.005$
No LIPUS (37.0 & 43.4 °C )	$\overline{{R}_{a}^{2}}=0.962$	$\overline{{R}_{a}^{2}}=0.991$	$\overline{{{\varvec{R}}}_{{\varvec{a}}}^{2}}=$**0.997**	$\overline{{R}_{a}^{2}}=0.991$
With LIPUS (37.0 °C )	${K}_{0}=0.880$ $RMSE=31.71$ $SSE=2.518$	${K}_{1}=0.038$ $RMSE=0.057$ $SSE=0.016$	${K}_{H}=8.931$ $RMSE=2.564$ $SSE=32.86$	${K}_{KP}=7.00$ $RMSE=0.051$ $SSE=0.013$
With LIPUS (43.4 °C )	${K}_{0}=0.735$ $RMSE=5.634$ $SSE=158.7$	${K}_{1}=0.120$ $RMSE=0.218$ $SSE=0.191$	${K}_{H}=7.692$ $RMSE=1.826$ $SSE=16.67$	${K}_{KP}=33.3$ $RMSE=0.018$ $SSE=0.002$
With LIPUS (37.0 & 43.4 °C )	$\overline{{R}_{a}^{2}}=0.936$	$\overline{{R}_{a}^{2}}=0.953$	$\overline{{R}_{a}^{2}}=0.985$	$\overline{{{\varvec{R}}}_{{\varvec{a}}}^{2}}=$**0.992**

Significant values are in bold.

**Figure 6:**
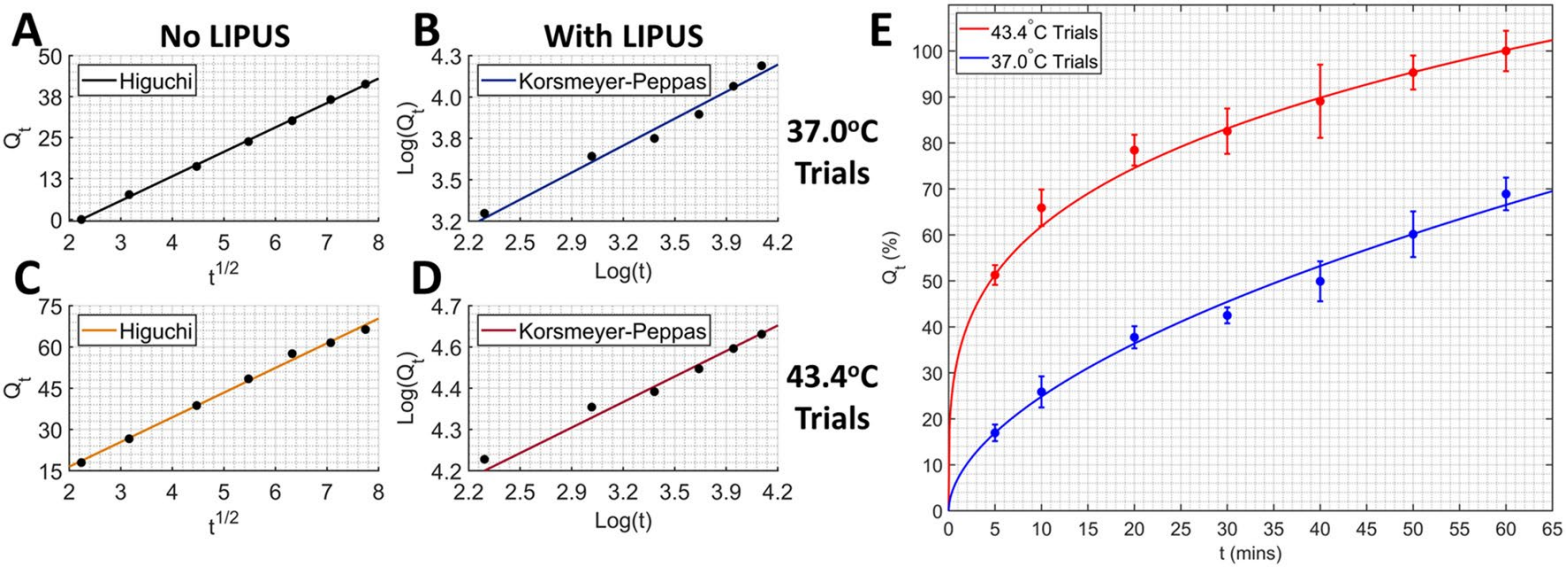
The highest agreement release kinetics linear fit for (**A**) no LIPUS at 37.0 °C, (**B**) with LIPUS at 37.0 °C, (**C**) no LIPUS at 43.4 °C, and (**D**) with LIPUS at 43.4 °C trials. All time values are in units of minutes. (**E**) DOX release for LIPUS 37.0 °C and 43.4 °C trials with their respective K–P fits.

The Higuchi kinetics fits for thermal release trials, and the K–P kinetics fits for LIPUS release trials, are shown in Fig. 6A–D. The release constant and release exponent for the K–P fits were extracted from Fig. 6B,D, with the linear fit slope taken as $n$, and the y-intercept taken as $\mathrm{log}({K}_{KP})$. The K–P release equation for LIPUS trials at both temperatures tested are provided below:

11 $${Q}_{t}\left(37.0 \, ^\circ \mathrm{C}\right)=\left(7.00\right){t}^{\left(0.550\right)}$$

12 $${Q}_{t}\left(43.4 \, ^\circ \mathrm{C}\right)=\left(33.3\right){t}^{\left(0.269\right)}$$

Here ${Q}_{t}\left(37.0^\circ \mathrm{C}\right)$ and ${Q}_{t}\left(37.0^\circ \mathrm{C}\right)$ are unitless percent cumulative DOX release values. Equations (11) and (12) were fitted to the LIPUS release data for both temperatures and are presented in Fig. 6E.

### Quantifying thermal and non-thermal release contributions

To quantify the contributions of thermal and non-thermal mechanisms of LIPUS-induced DOX release, ${\omega }_{T}$ and ${\omega }_{NT}$ were calculated for the 37.0 °C and 43.4 °C trials using Eqs. (7) and (8). For 37.0 °C trials, thermal mechanisms account for 60 ± 5% of release, while non-thermal mechanisms account for 40 ± 7%. For 43.4 °C trials, thermal mechanisms account for 66 ± 4% of the release, while non-thermal mechanisms account for 34 ± 5%. Calculated weighting factors are presented in Table 3.

**Table 3:** Calculated thermal and non-thermal weighting factors for 37.0 °C and 43.4 °C trials.

Variable water bath temperature	DOX release contributions	Value (%)
37.0 °C	Thermal (${\omega }_{T}$)	60 ± 5
	Non-thermal (${\omega }_{NT}$)	40 ± 7
43.4 °C	Thermal (${\omega }_{T}$)	66 ± 4
	Non-thermal (${\omega }_{NT}$)	34 ± 5

All values are averaged over six experiments with uncertainty taken as the standard error.

### LIPUS acoustic field simulation

**Figure 7:**
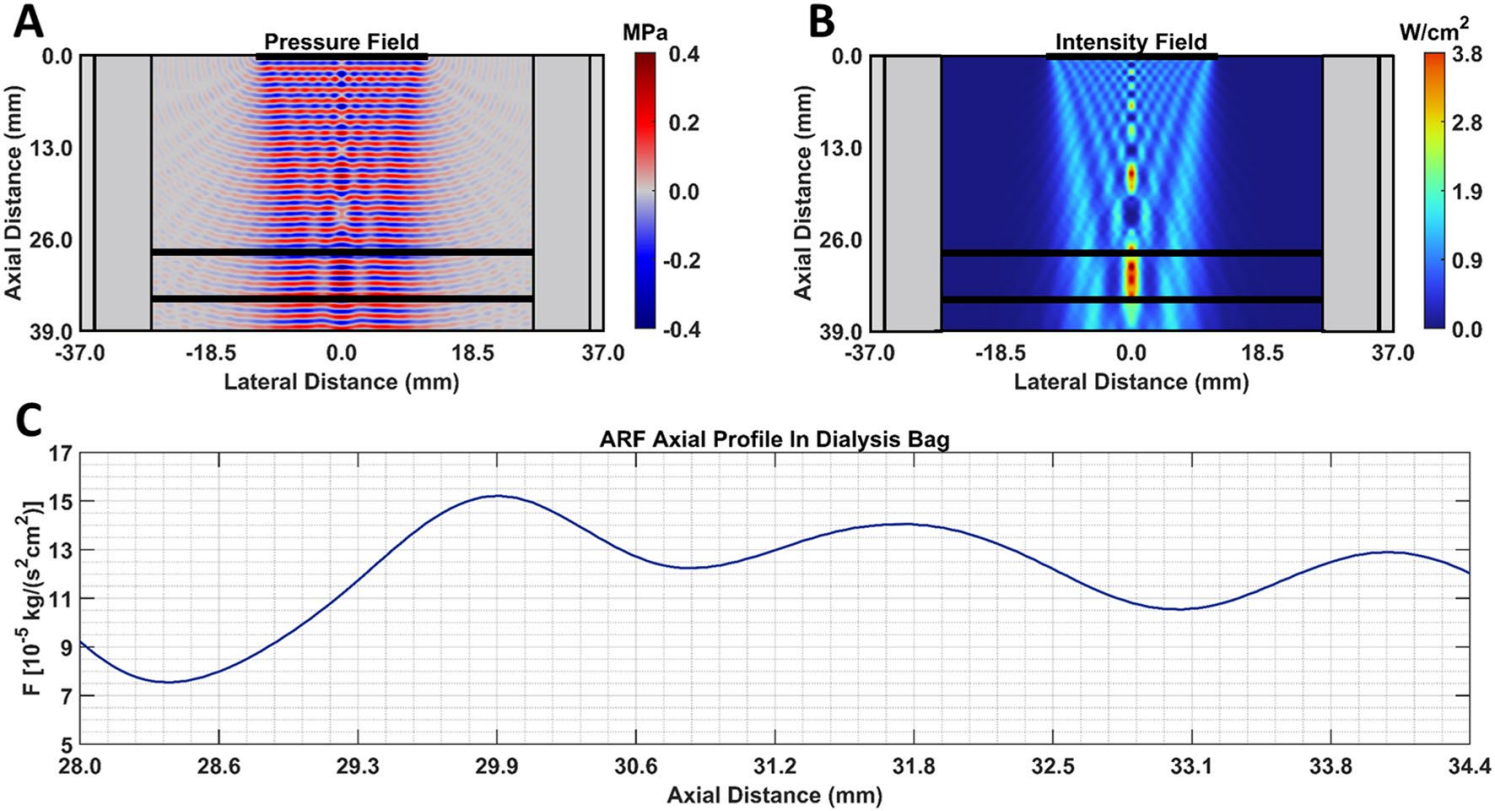
COMSOL simulated 2D (**A**) pressure and (**B**) time-averaged intensity fields inside the dialysis chamber. The LIPUS element, dialysis membrane, acrylic chamber walls, and nylon closure clips are marked for reference. (**C**) The ARF axial profile through the center of the dialysis membrane.

The COMSOL pressure acoustics simulation produced 2D axisymmetric pressure and intensity fields inside the dialysis chamber, as seen in Fig. 7A,B. The axial ARF profile through the center of the dialysis membrane was calculated using Eq. (9), as seen in Fig. 7C. A maximum ARF value of 15.2 × 10^–5^ kg/s^2^cm^2^ and an average ARF value of 12.0 × 10^–5^ kg/s^2^cm^2^ were calculated inside the dialysis bag. The maximum time-averaged intensity inside the dialysis membrane was 3.8 W/cm^2^, while an acoustic pressure range of − 0.4 MPa to 0.4 MPa was calculated. Given a maximum negative pressure of -0.4 MPa and a center frequency of 1 MHz, the $\mathrm{MI}$ was calculated as 0.4 using Eq. (10).

## Discussion

Overall, this work serves as the first drug release kinetics study for ultrasound-triggered drug release from GNP drug carriers. DOX release was successfully achieved from the surface of GNP drug carriers for thermal release only (no LIPUS) and LIPUS release (thermal and non-thermal release) trials at two different temperatures. The 37.0 °C LIPUS trial (Fig. 4A) could be considered an approximation of a non-thermal-only release trial. There is no significant heating of the GNPs above the clinical background temperature (37.0 °C), and any difference between the LIPUS and thermal release trials is due to the addition of non-thermal LIPUS-GNP interactions triggering DOX release. This is a good representation of how non-thermal LIPUS interactions trigger DOX release even without added hyperthermia effects. In the 43.4 °C LIPUS trial (Fig. 4B), the GNPs are heated to the hyperthermia temperature range, increasing DOX release due to the combination of thermal and non-thermal release mechanisms. In this trial the temperature in the dialysis membrane follows a similar temperature profile to what would be seen in clinical use (Fig. 5B). The GNPs are heated to the hyperthermia temperature regime and reach 43.4 °C 3.5 min after the start of the trial, then gradually cool to 37.0 °C 13 min after the 5-min LIPUS exposure. Any DOX release triggered in this trial is indicative of what would be seen in our previous ex vivo studies of LIPUS-triggered DOX release^8^, as well as what would be expected in future in vivo applications of our established 5-min 8.4 W 50% duty cycle LIPUS drug delivery system.

By comparing thermal and LIPUS release trials, thermal and non-thermal weighting factors for LIPUS release were calculated (Table 3). The significant contribution of non-thermal mechanisms to DOX release could account for the change in release rate trends between the thermal (no LIPUS) and LIPUS release trials. Furthermore, the sizeable non-thermal weighting factors suggest that non-thermal mechanisms play an important role in DOX release under LIPUS exposure, supporting our previous studies^8^. To study these non-thermal contributions, the finite element simulation of the LIPUS acoustic field was used. A 3.8 W/cm^2^ maximum in LIPUS acoustic intensity was found at the center of the dialysis bag. The calculated MI value of 0.40 was far below the 1.9 threshold for diagnostic imaging, suggesting that cavitation is not a dominant non-thermal effect in this style of drug delivery^44^. Therefore, ARF is expected to be the driving mechanism of non-thermal DOX release. An average ARF value of 12.0 × 10^–5^ kg/s^2^cm^2^ was calculated along the axial profile of the dialysis bag, which was slightly higher than the average value found in our previous ex vivo trials^8^. However, this could be due to the low absorption coefficient of the water domain traversed by the LIPUS beam before reaching the DOX-loaded GNPs in the dialysis membrane setup. Acoustic streaming is also an important non-thermal interaction to consider, as it could heavily influence circulation of released DOX from the dialysis membrane into the surrounding water domain.

As seen in the thermal release trials (no LIPUS), DOX release from the surface of GNPs followed a single phase of exponential release. With the addition of non-thermal release mechanisms in the LIPUS trials, DOX release kinetics appear to change to follow a multi-phase release trend. In both LIPUS trials, there was an initial phase of significant burst release. 51% and 17% of all DOX release was achieved at the end of the 5-min LIPUS exposure in the 43.4 °C and 37.0 °C trials, respectively. Burst release continued until approximately 20 min, with 38% release in the 37.0 °C and 78% release in the 43.4 °C trials achieved at that time. This was followed by constant release between 20 and 30 min and a higher rate release from 30 to 60 min.

When fitting release kinetic models to the DOX release data, the thermal release (no LIPUS) trials showed the highest agreement with the Higuchi model, which is consistent with the established literature^31,32^. High agreement with the Higuchi model suggests Fickian diffusion as the mechanism of DOX release, where release from the citrate surface layer occurs along the concentration gradient with a constant release rate over time^25,48–50^. However, with the addition of non-thermal release mechanisms in LIPUS-triggered DOX release trials, the release kinetics shifts from a Higuchi to a K–P best fit. A K–P release exponent of $n=0.550$ was extracted using only DOX release data in the 37.0 °C LIPUS trial, as a majority of the measured values meet the suggested ${Q}_{t}<60\%$ criteria^26^. A release exponent within 0.45 < n < 0.85 is indicative of anomalous (non-Fickian) diffusion, a dynamic process characterized by changing diffusion patterns over time^51^. Non-Fickian diffusion implies competing unknown release mechanisms beyond simple diffusion driving drug release, and typically includes a burst release phase followed by constant release^25^. The dynamic release phases can be attributed to alterations in pressure, temperature, and mechanical stress^52^, all of which are present in our LIPUS trials. The mechanisms of K–P drug release can be attributed to complex processes such as swelling, erosion or desorption of the citrate surface layer, which competes with diffusion to result in dynamic drug release profiles that deviate from typical Fickian diffusion. The K–P kinetic model is commonly used to model release from polymeric systems; however, the strong correlation with our LIPUS trials suggests that the release of DOX from the citrate layer on the GNP surface could be modeled similarly to a matrix or polymeric nanoparticle which undergoes degradation^26^. A similar conclusion was reached by England et al.^31^, who studied the release kinetics of citrate-capped GNPs.

Anomalous diffusion of the citrate surface layer under LIPUS exposure could explain the rapid burst release phase seen in the LIPUS release trials (Fig. 4). However, the exact mechanism of ultrasound-triggered drug release is complex and difficult to attribute to a single factor. In our green synthesis, the GNPs are coated with several layers of citrate and DOX. It is well known that citrate binds weakly on GNPs^53^, and the outmost layers are more loosely bound than the inner layers. This is primarily due to the contributions of steric repulsion between citrate layers^54^. In our case, both citrate and DOX adhere to GNPs by non-covalent electrostatic interactions^8,27,35^, with DOX mostly adhered to GNPs by hydrophobic functional groups^55^. Thus, we believe that when the GNPs are subjected to ultrasound waves, the thermal and non-thermal effects induced by LIPUS can remove the outermost loosely bound DOX and citrate layers, contributing to burst release. As determined in our previous work^27^, the citrate surface layer is critical to maintaining colloidal stability, so any damage to the surface layer will lead to rapid DOX release and GNP aggregation. Furthermore, ARF and acoustic streaming are expected to contribute to rapid clearance of released DOX from the dialysis bag under LIPUS exposure during the first 5-min of the LIPUS release trial. This further contributes to the measured burst release of DOX in the surrounding water domain. Burst release is also compounded by simple degradation and disruption of inner layer non-covalent bonds between the DOX and trisodium citrate on the GNP surface due to water bath heating, which further releases the DOX payload^55^. Burst release of chemotherapeutic drugs from GNPs has also been observed by Thambiraj et al.^32^ and England et al.^31^, who contributed burst release to the degradation of citrate non-covalent bonds. In our work, burst release is followed by dynamic diffusion driven by thermal mechanisms of release in the second and third release phases^32^. The rapid release of DOX in the initial burst phase under LIPUS exposure suggests that we can rapidly release our chemotherapeutic drug on-demand at the target site. This can prove advantageous in many chemotherapeutic drug delivery applications^56^. Overall, we contribute the mechanism of DOX release from the surface of the GNPs under LIPUS exposure may to a combination of desorption of the surface-bound DOX, erosion or swelling of the citrate surface layer and constant non-Fickian diffusion (anomalous diffusion) mechanisms.

Since the 43.4 °C LIPUS trial (Fig. 6E) follows a realistic temperature profile at the GNP surface, we propose that Eq. (12) could be applied to predict DOX release from the surface of GNPs as a function of time for our established 5-min LIPUS drug delivery system^8,27,35^. In future work, the fitted K–P model could be coupled with drug transport equations to simulate the more complex dynamics of DOX transport after LIPUS-triggered release in a solid tumour geometry, as there is a gap in knowledge of drug release parameters in such models^24^.

It is important to note that this study’s fitted release kinetics model only applies to citrate-layered GNPs and an unfocused LIPUS beam at 8.4 W (50% duty cycle). If other total acoustic powers or other therapeutic ultrasound transducers, such as focused ultrasound, are to be used to trigger drug release, DOX release kinetics could change due to the difference in the ARF and cavitation dominance. Furthermore, if the ultrasound exposure time is changed from the fixed 5-min LIPUS exposure time, a new kinetic study will need to be performed for DOX release under the set ultrasound exposure. Furthermore, including the finite element model allows us to study the effect of the dialysis membrane plastic clips and the dialysis chamber on the acoustic field. One limitation of our experimental method is that there is an unavoidable reflection of the LIPUS acoustic waves from the sides of the dialysis chamber. In a more realistic in vivo setup, we would not expect to see this reflection, and therefore the acoustic intensity driving ARF could potentially change. Lastly, hydrophobic anticancer drugs could be tested to determine if hydrophobic drugs affect GNP release kinetics under ultrasound exposure.

## Conclusion

This work studied DOX release kinetics from the surface of GNP drug carriers in a dialysis membrane setup. Four DOX release trials were performed with and without LIPUS exposure at 37.0 °C and 43.4 °C. Weighting factors were calculated for the contributions of thermal and non-thermal mechanisms of LIPUS-induced DOX release, with non-thermal mechanisms accounting for 40 ± 7% and 34 ± 5% of DOX release in the 37.0 °C and 43.4 °C trials, respectively. DOX release in LIPUS trials followed a multi-phase release temporal profile. An initial burst release phase was observed, followed by two release stages that we hypothesized could be due to non-Fickian diffusion across the dialysis membrane and constant thermal release. DOX release kinetics were then studied using zero-order, first-order, Higuchi, and K–P models for all four trials. DOX release in thermal-only trials (no LIPUS) was found to show the best agreement with Higuchi kinetics for both temperatures tested. In contrast, LIPUS trials showed a shift towards K–P kinetics. The LIPUS-induced DOX release trials at 43.4 °C were considered a realistic kinetic model of our established 5-min LIPUS drug delivery system and fitted with the K–P release kinetics model. Here, a release exponent of $n=0.550$ was found with a release constant of ${K}_{KP}=7.00$. DOX release from the surface of the GNPs may be attributed to anomalous diffusion of the DOX and trisodium citrate surface layer and degradation of the DOX-citrate non-covalent bonds under LIPUS exposure. In summary, the introduction of LIPUS non-thermal interactions shifts the mechanism of DOX release from a Fickian (static) to a non-Fickian (dynamic) release profile, highlighting the complexity of the interplay between ultrasound and drug release from gold nanoparticle drug carriers. Lastly, the LIPUS time-averaged intensity was simulated inside the dialysis membrane to quantify the MI and ARF in the dialysis bag. It was concluded that ARF could be a driving mechanism of LIPUS-induced non-thermal DOX release.

## Data Availability

Data can be obtained from the corresponding author Jahan Tavakkoli upon reasonable request.
